# Plasma total fibroblast growth factor 23 levels are associated with acute kidney injury and mortality in children with acute respiratory distress syndrome

**DOI:** 10.1371/journal.pone.0222065

**Published:** 2019-09-05

**Authors:** Mark R. Hanudel, Matthew S. Zinter, Lucia Chen, Kinisha Gala, Michelle Lim, Mona Guglielmo, Tanaya Deshmukh, Sitaram Vangala, Michael Matthay, Anil Sapru

**Affiliations:** 1 Department of Pediatrics, David Geffen School of Medicine at UCLA, Los Angeles, CA, United States of America; 2 Department of Pediatrics, UCSF School of Medicine, San Francisco, CA, United States of America; 3 Department of Medicine, David Geffen School of Medicine at UCLA, Los Angeles, CA, United States of America; 4 Department of Medicine, UCSF School of Medicine, San Francisco, CA, United States of America; National Yang-Ming University, TAIWAN

## Abstract

Acute respiratory distress syndrome (ARDS) has high rates of mortality and multisystem morbidity. Pre-clinical data suggest that fibroblast growth factor 23 (FGF23) may contribute to pulmonary pathology, and FGF23 is associated with mortality and morbidity, including acute kidney injury (AKI), in non-ARDS cohorts. Here, we assess whether FGF23 is associated with AKI and/or mortality in a cohort of 161 pediatric ARDS patients. Plasma total (intact + C-terminal) FGF23 and intact FGF23 concentrations were measured within 24 hours of ARDS diagnosis (Day 1), and associations with Day 3 AKI and 60-day mortality were evaluated. 35 patients (22%) developed AKI by 3 days post-ARDS diagnosis, and 25 (16%) died by 60 days post-ARDS diagnosis. In unadjusted models, higher Day 1 total FGF23 was associated with Day 3 AKI (odds ratio (OR) 2.22 [95% confidence interval (CI) 1.62, 3.03], p<0.001), but Day 1 intact FGF23 was not. In a model adjusted for demographics and disease severity, total FGF23 remained associated with AKI (OR 1.52 [95% CI 1.02, 2.26], p = 0.039). In unadjusted models, both higher Day 1 total and intact FGF23 were associated with 60-day mortality (OR 1.43 [95% CI 1.07, 1.91], p = 0.014; and OR 1.44 [95% CI 1.02, 2.05], p = 0.039, respectively). In the adjusted model, only total FGF23 remained associated with 60-day mortality (OR 1.62 [95% CI 1.07, 2.45], p = 0.023). In a subgroup analysis of patients with Day 1 plasma IL-6 concentrations available, inflammation partially mediated the association between total FGF23 and AKI. Our data suggest both inflammation-dependent and inflammation-independent associations between total FGF23 and clinical outcomes in pediatric ARDS patients.

## Introduction

Acute respiratory distress syndrome (ARDS) is defined as the presence of hypoxia in the context of a new lung infiltrate occurring within seven days of a known insult [1]. In children, ARDS is accompanied by high mortality rates—with an estimated overall mortality rate of 24% [2]—and extra-pulmonary comorbidities, including renal dysfunction, which occurs commonly and contributes substantially to morbidity and mortality [3–5]. ARDS may be precipitated by a pulmonary insult, such as pneumonia or aspiration (direct ARDS), or by a non-pulmonary insult, such as sepsis or transfusion reaction (indirect ARDS), resulting in non-cardiogenic pulmonary edema and massive pulmonary inflammation [1, 6, 7].

One factor that may contribute to pulmonary inflammation is fibroblast growth factor 23 (FGF23). FGF23 is a predominantly bone-derived hormone that acts on the kidney and physiologically functions to maintain phosphate homeostasis; however, FGF23 can also have pathologic, “off-target” effects. Specifically, FGF23 can induce cardiomyocyte hypertrophy [8], impair neutrophil function [9], and stimulate hepatic secretion of the inflammatory cytokines interleukin-6 (IL-6) and C-reactive protein [10], as has been demonstrated in *in vitro* and murine studies. Recently, it has also been shown that FGF23 can stimulate IL-6 release from cultured bronchial epithelial cells [11], suggesting a possible pro-inflammatory role of FGF23 in pathologic pulmonary conditions such as ARDS.

Therefore, FGF23 may induce inflammation, but interestingly, inflammation also affects FGF23. Inflammation promotes FGF23 proteolysis, resulting in increased levels of FGF23 fragments [12]. In the circulation, concentrations of both total FGF23 (intact FGF23 + C-terminal FGF23) and intact FGF23 alone can be measured. Whereas intact FGF23 is known to be biologically active, the effects of FGF23 fragments remain unclear.

In many human cohorts, higher circulating concentrations of total FGF23 and/or intact FGF23 have been associated with adverse clinical outcomes, including all-cause mortality [13–16], cardiovascular morbidity [8, 17–20], progression of chronic kidney disease [14, 21, 22], development of acute kidney injury (AKI) [23–28], and infection-related hospitalization [29]. However, whether FGF23 levels are associated with poor clinical outcomes in pediatric ARDS is unknown, and characterization of the FGF23 profile in this population may improve risk stratification and better define the pathophysiology of this heterogeneous clinical condition [30]. Therefore, in the current study, we assessed whether circulating total and intact FGF23 levels are associated with the development of AKI and/or mortality in a multicenter cohort of pediatric ARDS patients.

## Methods

### Study subjects

Data were collected from a multicenter observational study of pediatric intensive care unit patients with ARDS admitted between 2008 and 2016. Subjects were enrolled in five academic pediatric intensive care units: Children’s Hospital Los Angeles; Children’s Hospital Central California; American Family Children’s Hospital, University of Wisconsin-Madison; and the University of California San Francisco (UCSF) Benioff Children’s Hospitals in Oakland and San Francisco. The study was approved by the individual Institutional Review Boards at participating centers.

Pediatric patients with bilateral chest X-ray infiltrates, receiving respiratory support in the form of continuous positive airway pressure (CPAP), bilevel positive airway pressure (BiPAP), or invasive positive pressure ventilation, were screened for eligibility. Guardians were approached for informed written consent if the patients met the American-European Consensus Conference definition of Acute Lung Injury/Acute Respiratory Distress Syndrome [31]. Chest x-ray results used for diagnosing ARDS were based on interpretations performed by site investigators. Exclusion criteria included patients <1 month of age, <36 weeks corrected gestational age, >18 years of age, and/or with a documented Do Not Resuscitate or Do Not Intubate order at the time of screening.

### Data collection

**Fig 1:**
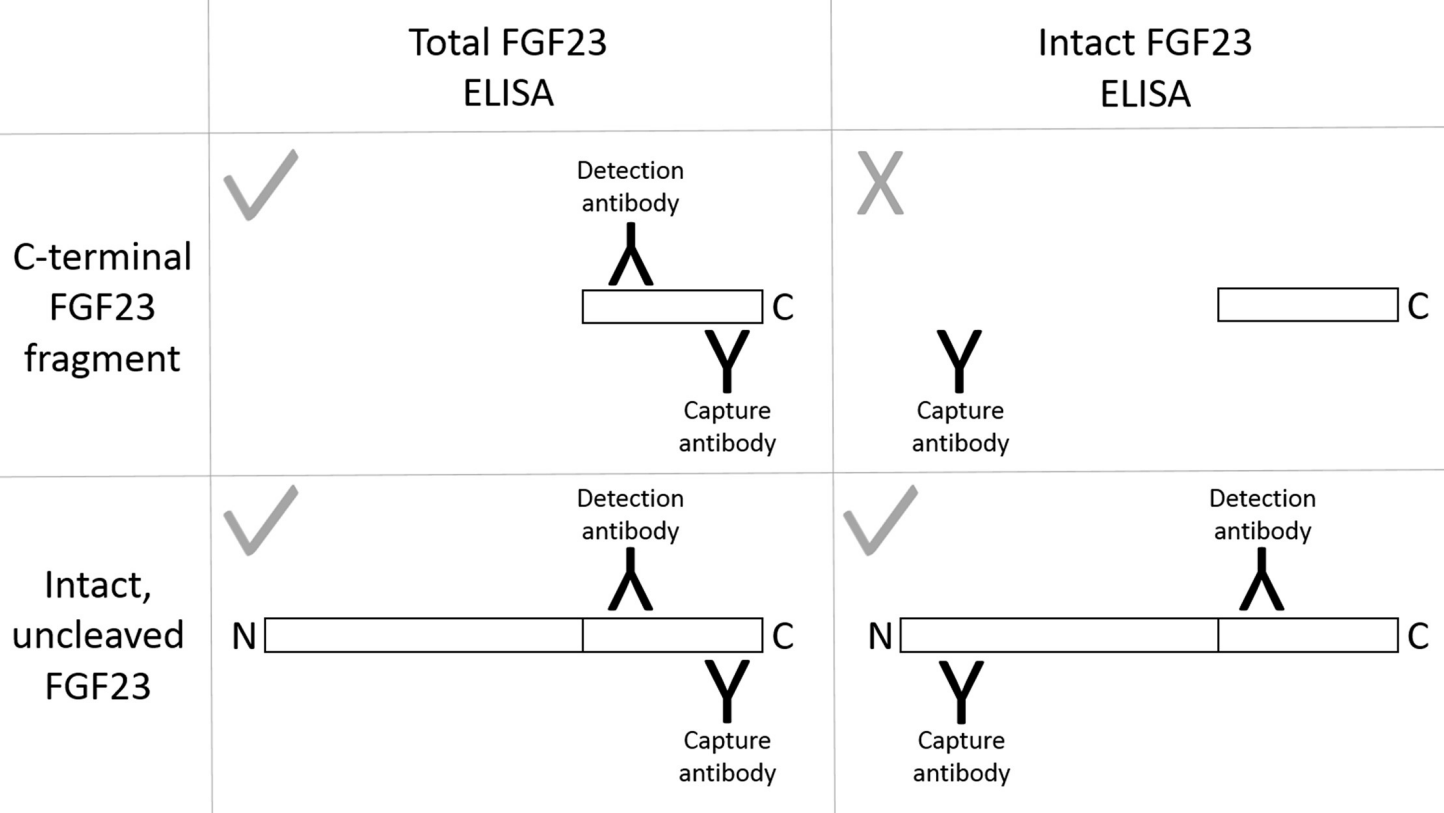
The total FGF23 and intact FGF23 enzyme-linked immunosorbent assays (ELISA). Whereas the total fibroblast growth factor 23 (FGF23) ELISA detects both the full-length, intact protein and its C-terminal proteolytic fragments, the intact FGF23 assay detects only the full-length form of the hormone.

Demographic data, anthropometric data, and causes of lung injury were obtained from the medical record. On the day of ARDS diagnosis (Day 1), plasma was collected for measurement of total (intact + C-terminal) FGF23 and intact FGF23, assessed with ELISA kits (Quidel, San Diego, CA). FGF23 can be proteolytically cleaved into fragments. The total FGF23 assay uses a C-terminal capture antibody and a C-terminal detection antibody, both of which recognize epitopes distal to the FGF23 cleavage site. Thus, the total FGF23 assay detects both full-length, intact FGF23 protein and C-terminal FGF23 proteolytic fragments. Conversely, the intact FGF23 assay uses an N-terminal capture antibody and a C-terminal detection antibody, thus detecting only full-length FGF23 (**Fig 1**) [32]. As the human total FGF23 assay measures concentrations in RU/ml, but the human intact FGF23 assay measures concentrations in pg/ml, direct calculation of C-terminal FGF23 fragment concentrations is not possible with these assays.

Serum creatinine was assessed daily, and glomerular filtration rate (GFR) was estimated using the revised Schwartz equation [33]. Consistent with the pediatric Risk, Injury, Failure, Loss, End-Stage (pRIFLE) criteria [34], AKI was defined as a decrease in estimated GFR (eGFR) of ≥50%. As baseline (prior to Day 1) serum creatinine measurements were not available, a baseline eGFR of 120 mL/min/1.73 m^2^ was assumed, as has been done in previous analyses of AKI in this cohort [35]. As a measurement of disease severity, Pediatric Risk of Mortality (PRISM) 3 scores were calculated [36]. PRISM 3 is a score developed specifically for mortality prediction, utilizing the worst values of 17 laboratory and vital sign parameters from the first 24 hours of intensive care [36]. PaO_2_/FiO_2_ (P/F) ratios were also calculated. For those subjects without recorded arterial blood gas data, with pulse oximetry saturations between 80 and 97%, and for whom FiO_2_ was available, predicted P/F was calculated using the saturation/FiO_2_ ratio [37]. A subgroup of patients had Day 1 plasma IL-6 concentrations available, which were measured with a Luminex multiplex ELISA (Myriad RBM, Austin, TX).

### Statistical analysis

All analyses were performed using STATA statistical software, version 13.1 (StataCorp, College Station, TX). Continuous data are presented as medians and interquartile ranges (IQR), and categorical data are presented as frequencies (percentage). Statistical tests used to compare data between groups were the Wilcoxon rank-sum test for continuous variables and the chi-squared test for categorical variables. Logistic regression modeling was performed to assess whether FGF23 levels at the time of diagnosis were associated with AKI at 3 days post-diagnosis or with mortality at 60 days post-diagnosis. Given skewed data distributions, natural log transformed FGF23 levels were used in the regression models. In the Day 3 AKI models, covariates included demographics (age, sex); measures of disease severity (P/F ratio, PRISM score); and the presence or absence of AKI at the time of diagnosis. In the 60-day mortality models, covariates included demographics (age, sex); measures of disease severity (P/F ratio, PRISM score); and the presence or absence of AKI. These models were fitted using a complete cases approach, and numbers of included patients are reported for each regression model. Separate analyses were performed including plasma IL-6 as a covariate, due to only a subset of patients having non-missing data. Given skewed data distributions, natural log transformed IL-6 was used in the regression models. Correlations between IL-6 and FGF23 concentrations were assessed with Spearman’s rank correlation coefficients. Also, in this subset, we performed mediation analysis [38, 39] to quantify the degree to which variation in IL-6 concentrations mediated associations between FGF23 levels and clinical outcomes. In all analyses, a two-sided p-value <0.05 was considered significant.

## Results

### Cohort characteristics

The cohort included 161 pediatric ARDS patients, 57% male, 70% Caucasian, with a median age of 4.4 [IQR 1.1, 11.7] years (**Table 1**). The major risk factors for ARDS were pneumonia (60%), sepsis (19%), and aspiration (6%). At the time of ARDS diagnosis (Day 1), the median plasma total FGF23 level in this cohort was markedly elevated at 223 [IQR 114, 774] RU/ml. (In healthy children, median total FGF23 concentrations range from 50 to 105 RU/ml, depending on age [40].) Contrastingly, the Day 1 median intact FGF23 level was not elevated (23 [IQR 11, 60] pg/ml). (In healthy children, the median intact FGF23 concentration is 35 [range 9–120] pg/ml [41].) Of the 161 subjects, 35 (22%) had AKI on Day 3 post-ARDS diagnosis (**S1 Table**), and 25 (16%) died by 60 days post-ARDS diagnosis.

**Table 1:** Cohort characteristics at the time of ARDS diagnosis, stratified by the presence/absence of Day 3 AKI and stratified by 60-day mortality.

Variable	All (n = 161)	No Day 3 AKI (n = 126, 78%)	Day 3 AKI (n = 35, 22%)	p value	Survived (n = 136, 84%)	Deceased (n = 25, 16%)	p value
Age (years)	4.4 (1.1, 11.7)	5.9 (1.5, 11.8)	2.2 (0.4, 11.3)	0.21	3.4 (1.1, 11.3)	10.4 (2.9, 13.9)	0.11
Sex (% male)	92 (57%)	71 (56%)	21 (60%)	0.70	73 (54%)	19 (76%)	0.038
Race:				0.21			1.00
Caucasian/white	112 (70%)	91 (72%)	21 (60%)		95 (70%)	17 (68%)	
African-American/black	14 (9%)	9 (7%)	5 (14%)		12 (9%)	2 (8%)	
Asian/Pacific Islander	13 (8%)	8 (6%)	5 (14%)		11 (8%)	2 (8%)	
Other	22 (14%)	18 (14%)	4 (11%)		18 (13%)	4 (16%)	
ARDS etiology:				0.40			0.49
Pneumonia	96 (60%)	78 (62%)	18 (51%)		80 (58%)	16 (64%)	
Sepsis	30 (19%)	21 (17%)	9 (26%)		25 (18%)	5 (20%)	
Aspiration	9 (6%)	6 (5%)	3 (9%)		7 (5%)	2 (8%)	
Trauma	6 (4%)	5 (4%)	1 (3%)		5 (4%)	1 (4%)	
Transfusion	1 (1%)	1 (1%)	0 (0%)		1 (1%)	0 (0%)	
Other	18 (11%)	15 (12%)	3 (9%)		18 (13%)	0 (0%)	
Missing	1 (1%)	0 (0%)	1 (3%)		0 (0%)	1 (4%)	
Primary insult (% direct ARDS)	107 (66%)	86 (68%)	21 (62%)	0.48	89 (65%)	18 (75%)	0.36
Type of respiratory support:				0.67			0.65
Conventional mechanical ventilation	144 (89%)	112 (89%)	32 (91%)		121 (89%)	23 (92%)	
High frequency oscillatory ventilation	7 (4%)	6 (5%)	1 (3%)		6 (4%)	1 (4%)	
CPAP or BiPAP	10 (6%)	8 (6%)	2 (6%)		9 (7%)	1 (4%)	
Kidney function (% AKI)	35 (22%)	n/a	n/a	n/a	28 (21%)	7 (28%)	0.41
Day 1 PaO_2_/FiO_2_ ratio	153 (91, 228)	162 (93, 223)	129 (89, 243)	0.50	163 (92, 246)	135 (84, 172)	0.06
Day 1 oxygenation index	9 (5, 18)	8 (5, 16)	12 (6, 19)	0.25	8 (5, 16)	10 (8, 24)	0.07
Day 1 PRISM score	11 (6, 18)	10 (5, 17)	16 (10, 22)	0.001	11 (5, 17)	15 (9, 19)	0.04
Day 1 serum IL-6 (pg/ml) (n = 135)	75 (26, 227)	70 (18, 185)	138 (41, 1540)	0.004	72 (24, 222)	107 (28, 689)	0.21
Day 1 plasma total FGF23 (RU/ml)	223 (114, 774)	166 (89, 466)	944 (357, 6556)	<0.001	202 (100, 669)	529 (172, 1490)	0.017
Day 1 plasma intact FGF23 (pg/ml)	23 (11, 60)	21 (11, 41)	28 (13, 105)	0.09	21 (10, 43)	38 (13, 128)	0.037
Data presented as numbers (percentages) or medians (interquartile range).							

### Day 1 FGF23 levels and Day 3 AKI

**Fig 2:**
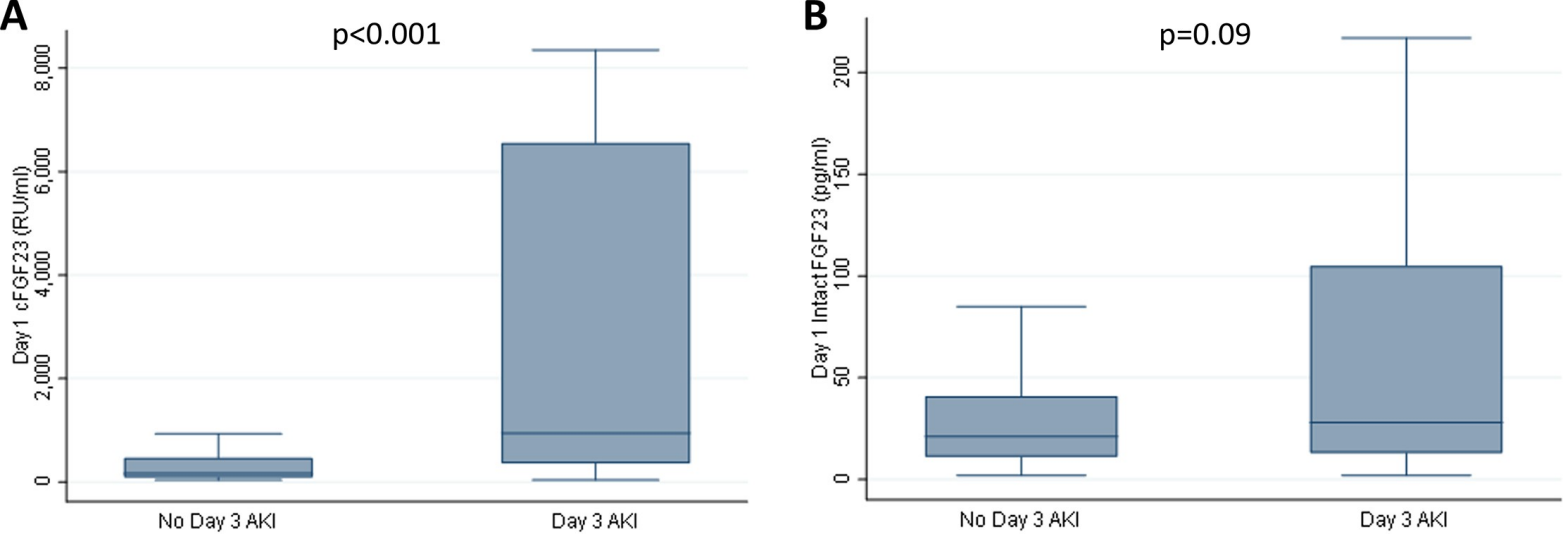
Day 1 plasma FGF23 concentrations stratified by the presence or absence of Day 3 AKI. Day 1 plasma total fibroblast growth factor 23 (cFGF23) concentrations were significantly higher in subjects who had acute kidney injury (AKI) on Day 3 than in those that did not (**Fig 2A**). Day 1 plasma intact FGF23 concentrations were not significantly different between the two groups (**Fig 2B**). Data are presented as medians and interquartile ranges, and the Wilcoxon rank-sum test was used to compare groups.

Cohort characteristics at the time of ARDS diagnosis, stratified by the presence or absence of Day 3 AKI, are shown in **Table 1**. Day 1 total FGF23 concentrations were significantly higher in subjects with Day 3 AKI than in those without Day 3 AKI (median 944 [IQR 357, 6556] RU/ml vs. median 166 [IQR 89, 466] RU/ml, p<0.001) (**Fig 2A**). However, Day 1 intact FGF23 levels did not significantly differ between the two groups (median 28 [IQR 13, 105] pg/ml in the AKI group vs. median 21 [IQR 11, 41] pg/ml in the non-AKI group, p = 0.09) (**Fig 2B**).

Higher Day 1 total FGF23 levels were significantly associated with the presence of Day 3 AKI (odds ratio (OR) 2.21 [95% confidence interval (CI) 1.61, 3.03], p<0.001) (**Table 2, Model 1**). The association persisted after adjustment for the presence of Day 1 AKI (OR 1.51 [95% CI 1.05, 2.17], p = 0.027) (**Table 2, Model 2**), and after further adjustment for Day 1 covariates (age, sex, P/F ratio, and PRISM score) (OR 1.52 [95% CI 1.02, 2.26], p = 0.039) (**Table 2, Model 3**). On the contrary, Day 1 intact FGF23 concentrations were not significantly associated with Day 3 AKI in any of the models.

**Table 2:** Multivariable logistic regression modeling, with a dependent variable of acute kidney injury (AKI) at three days post-ARDS diagnosis.

Dependent Variable	Independent Variable	Model 1	Model 2	Model 3
Day 3 AKI	Total FGF23	2.22 (1.62, 3.03), p<0.001	1.51 (1.05, 2.17), p = 0.027	1.52 (1.02, 2.26), p = 0.039
Day 3 AKI	Intact FGF23	1.28 (0.95, 1.72), p = 0.11	0.99 (0.69, 1.42), p = 0.95	1.02 (0.69, 1.52), p = 0.92

In these models, the independent variable is Day 1 plasma total FGF23 or intact FGF23, both of which were log-transformed to correct for skewness. Model 1 is unadjusted. Model 2 is adjusted for the presence or absence of Day 1 AKI. Model 3 is adjusted for Day 1 AKI, age, sex, P/F ratio, and PRISM score. Data shown are odds ratios and 95% confidence intervals. As only subjects with complete covariate data are included in the regression analysis, n = 150 for all models.

### Day 1 FGF23 levels and 60-day mortality

**Fig 3:**
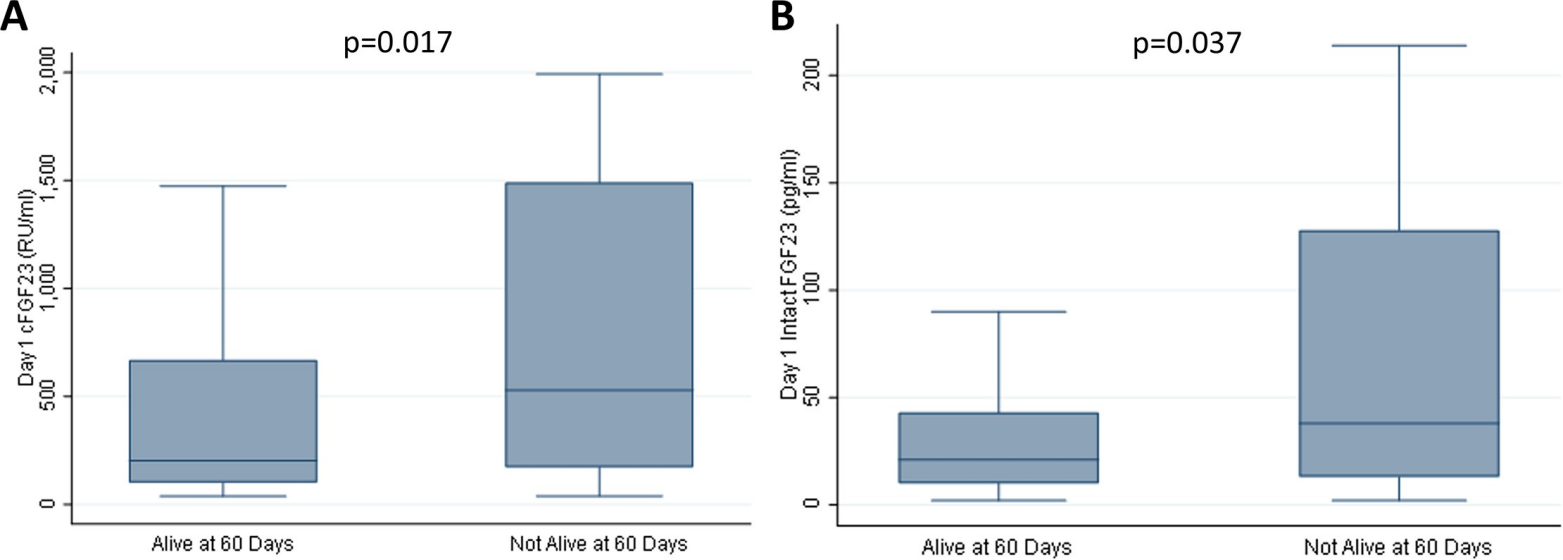
Day 1 plasma FGF23 concentrations stratified by 60-day mortality. Day 1 plasma total fibroblast growth factor 23 (cFGF23) concentrations (**Fig 3A**) and intact FGF23 concentrations (**Fig 3B**) were significantly higher in subjects who did not survive. Data are presented as medians and interquartile ranges, and the Wilcoxon rank-sum test was used to compare groups.

Cohort characteristics at the time of ARDS diagnosis, stratified by mortality, are shown in **Table 1**. Day 1 total FGF23 concentrations were significantly higher in subjects that died than in those that survived (median 529 [IQR 172, 1490] RU/ml vs. median 202 [IQR 100, 669] RU/ml, p = 0.017) (**Fig 3A**). Day 1 intact FGF23 concentrations were also significantly higher in subjects that died than in those that survived (median 38 [IQR 13, 128] pg/ml vs. median 21 [IQR 10, 43] pg/ml, p = 0.037) (**Fig 3B**).

Both higher Day 1 total FGF23 levels and higher Day 1 intact FGF23 levels were significantly associated with mortality at 60 days (OR 1.43 [95% CI 1.07, 1.91], p = 0.014; and OR 1.44 [95% CI 1.02, 2.05], p = 0.039, respectively) (**Table 3, Model 1**). After adjustment for Day 1 covariates (age, sex, presence/absence of AKI, P/F ratio, and PRISM score), Day 1 total FGF23 levels remained significantly associated with 60-day mortality (OR 1.62 [95% CI 1.07, 2.45], p = 0.023), but Day 1 intact FGF23 levels did not (OR 1.30 [95% CI 0.89, 1.90], p = 0.17) (**Table 3, Model 2**).

**Table 3:** Multivariable logistic regression modeling, with a dependent variable of mortality by 60 days post-ARDS diagnosis.

Dependent Variable	Independent Variable	Model 1	Model 2
Day 60 mortality	Total FGF23	1.43 (1.07, 1.91), p = 0.014	1.62 (1.07, 2.45), p = 0.023
Day 60 mortality	Intact FGF23	1.44 (1.02, 2.05), p = 0.039	1.30 (0.89, 1.90), p = 0.17

In these models, the independent variable is Day 1 plasma total FGF23 or intact FGF23, both of which were log-transformed to correct for skewness. Model 1 is unadjusted. Model 2 is adjusted for age, sex, the presence or absence of Day 1 AKI, P/F ratio, and PRISM score. Data shown are odds ratios and 95% confidence intervals. As only subjects with complete covariate data are included in the regression analysis, n = 150 for both models.

### Subgroup of patients with Day 1 IL-6 concentrations

Given that stronger associations were observed with total FGF23 than with intact FGF23, and that inflammation is known to increase total FGF23 out of proportion to intact FGF23 [12], we assessed how the addition of an inflammatory marker to our models evaluating total FGF23 affected the results. In a subgroup of patients (n = 135), Day 1 plasma IL-6 concentrations were available. In these patients, plasma IL-6 levels were markedly elevated compared to what is observed in healthy subjects [42]. Total FGF23 levels positively correlated with plasma IL-6 concentrations (Spearman’s rank correlation coefficient = 0.35, p<0.001), but intact FGF23 levels did not (Spearman’s rank correlation coefficient = 0.06, p = 0.45).

Regarding the outcome of AKI, the addition of IL-6 as a covariate to the subgroup adjusted model decreased the Day 1 total FGF23 OR from 1.42 (95% CI 0.95, 2.14; p = 0.09) to 1.26 (95% CI 0.81, 1.96; p = 0.31) (**Table 4**). In this adjusted model, plasma IL-6 concentrations were independently associated with Day 3 AKI (OR 1.59 [95% CI 1.13, 2.22], p = 0.007), similar to what has been observed in other analyses of this cohort [35]. Given the association between IL-6 and AKI, we performed mediation analysis to quantify the degree to which variation in IL-6 concentrations mediated the FGF23-AKI association. In this analysis, although the p-value for the indirect effect did not reach statistical significance (p = 0.12), likely contributed to by decreased sample size, it was estimated that 36.4% of the association between total FGF23 and AKI was explained by IL-6 (**S2 Table**), suggesting partial mediation.

**Table 4:** Multivariable logistic regression modeling in the subgroup of patients with Day 1 plasma IL-6 concentrations available.

Dependent Variable	Independent Variable	Model 1	Model 2	Model 3
Day 3 AKI	Total FGF23	1.99 (1.44, 2.75), p<0.001	1.42 (0.95, 2.14), p = 0.09	1.26 (0.81, 1.96), p = 0.31
Day 60 mortality	Total FGF23	1.27 (0.92, 1.75), p = 0.14	1.38 (0.90, 2.11), p = 0.14	1.35 (0.88, 2.09), p = 0.17

This subgroup includes 135 subjects. In these models, the dependent variable is acute kidney injury (AKI) at three days post-ARDS diagnosis or mortality by 60 days post-ARDS diagnosis. The independent variable is Day 1 plasma total FGF23, which was log-transformed to correct for skewness. Model 1 is unadjusted. Model 2 is adjusted for age, sex, the presence or absence of Day 1 AKI, P/F ratio, and PRISM score. Model 3 is further adjusted for log-transformed Day 1 plasma IL-6. Data shown are odds ratios and 95% confidence intervals.

Regarding the outcome of mortality, the addition of IL-6 as a covariate to the subgroup adjusted model did not alter the Day 1 total FGF23 OR (1.38 [95% CI 0.90, 2.11], p = 0.14 without IL-6 vs. 1.35 [95% CI 0.88, 2.09], p = 0.17 with IL-6) (**Table 4**).

## Discussion

In this study of a multicenter cohort of pediatric ARDS patients, we found that higher levels of plasma total FGF23, but not intact FGF23, measured at the time of ARDS diagnosis (Day 1) are associated with Day 3 AKI in unadjusted and adjusted models. We also found that higher levels of both Day 1 total and intact FGF23 are associated with 60-day mortality in unadjusted models, but in adjusted models, this association with mortality persists for only total FGF23.

FGF23 is a hormone that is primary secreted by osteocytes. Intracellularly, FGF23 production is regulated at both the transcriptional and post-translational stages. Prior to secretion, translated, full-length FGF23 protein can be cleaved by furin into N-terminal and C-terminal fragments, the biological functions of which are unclear. Therefore, what is secreted into the circulation is a mix of intact FGF23 protein and FGF23 proteolytic fragments, and post-translational cleavage mechanisms determine how much of the total FGF23 secreted from the cell is intact and how much is fragmented.

As the total FGF23 ELISA detects both intact and fragmented FGF23 in the circulation, it functions as a surrogate marker of total FGF23 translated and secreted. A profile of circulating FGF23 concentrations characterized by increased total FGF23 but normal intact FGF23 is consistent with complete coupling of increased FGF23 transcription with increased post-translational cleavage, resulting in increased circulating levels of FGF23 fragments. Notably, several stimuli couple increased FGF23 transcription with increased post-translational cleavage, including inflammation [12], iron deficiency [12, 43–45], erythropoietin [46–51], and parathyroid hormone [52], resulting in elevated concentrations of circulating total FGF23 but not intact FGF23. Indeed, in murine models of acute and chronic inflammation, bone *Fgf23* mRNA expression and circulating total FGF23 are increased, but circulating levels of intact FGF23 remain normal or near-normal [12]. As ARDS is an inflammatory condition, inflammation may have contributed to the differing results we observed for total FGF23 vs. intact FGF23.

In our cohort, circulating levels of total FGF23 were elevated, but not intact FGF23, consistent with what is observed in the presence of inflammation [12]. A subgroup of patients had plasma IL-6 concentrations—an inflammatory cytokine—available for analysis. Plasma IL-6 concentrations were elevated, and positively correlated with total FGF23 but not intact FGF23. In this subgroup, plasma IL-6 was independently associated with AKI, and the addition of IL-6 as a covariate to the adjusted regression model partially attenuated the association between total FGF23 and AKI, with mediation analysis demonstrating partial mediation by IL-6. Given the presence of partial mediation, these data suggest both inflammation-dependent and inflammation-independent associations between total FGF23 and AKI. Regarding possible inflammation-dependent effects, previous studies have demonstrated that higher circulating IL-6 levels are independently associated with AKI [35, 53–55], and that IL-6 may play a role in AKI pathophysiology [56]. Regarding possible inflammation-independent effects, the pathophysiologic mechanisms related to high concentrations of FGF23 fragments remain to be elucidated. Although several conditions result in high FGF23 fragment concentrations, studies assessing the direct effects of these fragments are limited. However, one recent study provides some data demonstrating a direct adverse effect of the C-terminal FGF23 fragment on cardiomyocytes [57].

In the limited subgroup of patients with both IL-6 and FGF23 levels, the addition of IL-6 as a covariate to the adjusted regression model examining the association of total FGF23 with mortality did not alter the odds ratio, suggesting inflammation-independent effects. However, it should be noted that the p-value associated with this odds ratio did not reach statistical significance, possibly contributed to by decreased power associated with the smaller subgroup sample size.

In a similar study, Leaf et al assessed FGF23-mortality associations in the Validating Acute Lung Injury biomarkers for Diagnosis (VALID) cohort, which comprised 710 adult ARDS patients recruited from a single ICU [58]. In that study, the highest vs. lowest quartile of FGF23 concentrations at enrollment was independently associated with a significantly increased risk of both 60-day and 1-year mortality after adjustment for demographics, comorbidities, and measures of illness severity [58]. Although both total FGF23 and intact FGF23 levels were independently associated with mortality, the association effect sizes were stronger for total FGF23 than intact FGF23, similar to what we observed in our pediatric ARDS cohort. Of note, in the VALID cohort, circulating IL-6 concentrations were not available for analysis, so this marker of inflammation could not be added to the models. However, in another critically ill cohort, without ARDS, but in which plasma IL-6 levels were available, Leaf et al observed that both total FGF23 and intact FGF23 levels remained associated with mortality after adjustment for IL-6, suggesting inflammation-independent effects of FGF23 [58].

Although our study is novel in its assessment of FGF23 in the pediatric ARDS population, it has some limitations, including a lack of IL-6 concentrations in all patients and possible contributions from unmeasured ARDS-relevant factors. Another limitation is that we measured FGF23 concentrations only at the time of ARDS diagnosis; it is unknown whether changes in FGF23 over time are associated with clinical outcomes. Lastly, as this was an observational study, only associative data was generated, without providing any evidence of causality between predictor variables and clinical outcomes.

In conclusion, based on pre-clinical data suggesting that FGF23 may contribute to pulmonary pathology [11, 59, 60], we assessed whether circulating FGF23 levels are associated with clinical outcomes in a multicenter cohort of pediatric ARDS patients. Total (intact + C-terminal) FGF23, but not intact FGF23 alone, was associated with AKI in unadjusted and adjusted models. Total and intact FGF23 were associated with mortality in unadjusted models, but only total FGF23 was associated with mortality in an adjusted model. In a subset of patients, plasma IL-6 concentrations correlated with total FGF23 but not intact FGF23, and partially mediated the association between total FGF23 and AKI, suggesting both inflammation-dependent and inflammation-independent effects. Building on the findings of previous studies, this study further demonstrates the robustness and utility of total FGF23 as a biomarker of adverse clinical outcomes in critically ill patient populations, potentially helping to identify those at highest risk. Further mechanistic studies are required to assess the possible direct pathophysiologic effects of FGF23 fragments.

## Supporting information

S1 Table Numbers of subjects with and without acute kidney injury (AKI). (DOCX)

S2 Table Mediation analysis of interleukin-6 in the association between total FGF23 and acute kidney injury. All coefficients are adjusted for age, sex, the presence or absence of Day 1 AKI, P/F ratio, and PRISM score. The 95% confidence intervals for the indirect effects were determined after running 10,000 bootstrap samples. The estimated size of the mediated effect (proportion mediated) was calculated as the indirect effect divided by the total effect, multiplied by 100.
(DOCX)

## References

[1] Heidemann SM, Nair A, Bulut Y, Sapru A. Pathophysiology and Management of Acute Respiratory Distress Syndrome in Children. Pediatric clinics of North America. 2017;64(5):1017–37. Epub 2017/09/25. 10.1016/j.pcl.2017.06.004 . 28941533 PMC9683071

[2] Wong JJ, Jit M, Sultana R, Mok YH, Yeo JG, Koh J, et al Mortality in Pediatric Acute Respiratory Distress Syndrome: A Systematic Review and Meta-Analysis. Journal of intensive care medicine. 2017:885066617705109. Epub 2017/05/04. 10.1177/0885066617705109 . 28460591

[3] Flori HR, Glidden DV, Rutherford GW, Matthay MA. Pediatric acute lung injury: prospective evaluation of risk factors associated with mortality. American journal of respiratory and critical care medicine. 2005;171(9):995–1001. Epub 2004/12/25. 10.1164/rccm.200404-544OC . 15618461

[4] Erickson S, Schibler A, Numa A, Nuthall G, Yung M, Pascoe E, et al Acute lung injury in pediatric intensive care in Australia and New Zealand: a prospective, multicenter, observational study. Pediatric critical care medicine: a journal of the Society of Critical Care Medicine and the World Federation of Pediatric Intensive and Critical Care Societies. 2007;8(4):317–23. Epub 2007/06/05. 10.1097/01.pcc.0000269408.64179.ff . 17545931

[5] Liu KD, Glidden DV, Eisner MD, Parsons PE, Ware LB, Wheeler A, et al Predictive and pathogenetic value of plasma biomarkers for acute kidney injury in patients with acute lung injury. Critical care medicine. 2007;35(12):2755–61. Epub 2007/12/13. 18074478 PMC3293249

[6] Dolinay T, Kim YS, Howrylak J, Hunninghake GM, An CH, Fredenburgh L, et al Inflammasome-regulated cytokines are critical mediators of acute lung injury. American journal of respiratory and critical care medicine. 2012;185(11):1225–34. Epub 2012/03/31. 10.1164/rccm.201201-0003OC 22461369 PMC3373064

[7] Reiss LK, Schuppert A, Uhlig S. Inflammatory processes during acute respiratory distress syndrome: a complex system. Current opinion in critical care. 2018;24(1):1–9. Epub 2017/11/28. 10.1097/MCC.0000000000000472 . 29176329

[8] Faul C, Amaral AP, Oskouei B, Hu MC, Sloan A, Isakova T, et al FGF23 induces left ventricular hypertrophy. The Journal of clinical investigation. 2011;121(11):4393–408. Epub 2011/10/12. 10.1172/JCI46122 21985788 PMC3204831

[9] Rossaint J, Oehmichen J, Van Aken H, Reuter S, Pavenstadt HJ, Meersch M, et al FGF23 signaling impairs neutrophil recruitment and host defense during CKD. The Journal of clinical investigation. 2016;126(3):962–74. Epub 2016/02/16. 10.1172/JCI83470 26878171 PMC4767336

[10] Singh S, Grabner A, Yanucil C, Schramm K, Czaya B, Krick S, et al Fibroblast growth factor 23 directly targets hepatocytes to promote inflammation in chronic kidney disease. Kidney international. 2016;90(5):985–96. Epub 2016/07/28. 10.1016/j.kint.2016.05.019 27457912 PMC5065745

[11] Krick S, Helton ES, Hutcheson SB, Blumhof S, Garth JM, Denson RS, et al FGF23 Induction of O-Linked N-Acetylglucosamine Regulates IL-6 Secretion in Human Bronchial Epithelial Cells. Frontiers in endocrinology. 2018;9:708 Epub 2018/12/13. 10.3389/fendo.2018.00708 30538676 PMC6277595

[12] David V, Martin A, Isakova T, Spaulding C, Qi L, Ramirez V, et al Inflammation and functional iron deficiency regulate fibroblast growth factor 23 production. Kidney international. 2016;89(1):135–46. Epub 2015/11/05. 10.1038/ki.2015.290 26535997 PMC4854810

[13] Gutierrez OM, Mannstadt M, Isakova T, Rauh-Hain JA, Tamez H, Shah A, et al Fibroblast growth factor 23 and mortality among patients undergoing hemodialysis. The New England journal of medicine. 2008;359(6):584–92. Epub 2008/08/09. 10.1056/NEJMoa0706130 18687639 PMC2890264

[14] Isakova T, Xie H, Yang W, Xie D, Anderson AH, Scialla J, et al Fibroblast growth factor 23 and risks of mortality and end-stage renal disease in patients with chronic kidney disease. Jama. 2011;305(23):2432–9. Epub 2011/06/16. 10.1001/jama.2011.826 21673295 PMC3124770

[15] Arnlov J, Carlsson AC, Sundstrom J, Ingelsson E, Larsson A, Lind L, et al Higher fibroblast growth factor-23 increases the risk of all-cause and cardiovascular mortality in the community. Kidney international. 2013;83(1):160–6. Epub 2012/09/07. 10.1038/ki.2012.327 . 22951890

[16] Brandenburg VM, Kleber ME, Vervloet MG, Tomaschitz A, Pilz S, Stojakovic T, et al Fibroblast growth factor 23 (FGF23) and mortality: the Ludwigshafen Risk and Cardiovascular Health Study. Atherosclerosis. 2014;237(1):53–9. Epub 2014/09/10. 10.1016/j.atherosclerosis.2014.08.037 . 25200615

[17] Ix JH, Katz R, Kestenbaum BR, de Boer IH, Chonchol M, Mukamal KJ, et al Fibroblast growth factor-23 and death, heart failure, and cardiovascular events in community-living individuals: CHS (Cardiovascular Health Study). Journal of the American College of Cardiology. 2012;60(3):200–7. Epub 2012/06/19. 10.1016/j.jacc.2012.03.040 22703926 PMC3396791

[18] Arnlov J, Carlsson AC, Sundstrom J, Ingelsson E, Larsson A, Lind L, et al Serum FGF23 and risk of cardiovascular events in relation to mineral metabolism and cardiovascular pathology. Clinical journal of the American Society of Nephrology: CJASN. 2013;8(5):781–6. Epub 2013/01/22. 10.2215/CJN.09570912 23335040 PMC3641622

[19] Scialla JJ, Xie H, Rahman M, Anderson AH, Isakova T, Ojo A, et al Fibroblast growth factor-23 and cardiovascular events in CKD. Journal of the American Society of Nephrology: JASN. 2014;25(2):349–60. Epub 2013/10/26. 10.1681/ASN.2013050465 24158986 PMC3904568

[20] Mehta R, Cai X, Lee J, Scialla JJ, Bansal N, Sondheimer JH, et al Association of Fibroblast Growth Factor 23 With Atrial Fibrillation in Chronic Kidney Disease, From the Chronic Renal Insufficiency Cohort Study. JAMA cardiology. 2016;1(5):548–56. Epub 2016/07/20. 10.1001/jamacardio.2016.1445 27434583 PMC4992989

[21] Fliser D, Kollerits B, Neyer U, Ankerst DP, Lhotta K, Lingenhel A, et al Fibroblast growth factor 23 (FGF23) predicts progression of chronic kidney disease: the Mild to Moderate Kidney Disease (MMKD) Study. Journal of the American Society of Nephrology: JASN. 2007;18(9):2600–8. Epub 2007/07/28. 10.1681/ASN.2006080936 . 17656479

[22] Portale AA, Wolf MS, Messinger S, Perwad F, Juppner H, Warady BA, et al Fibroblast Growth Factor 23 and Risk of CKD Progression in Children. Clinical journal of the American Society of Nephrology: CJASN. 2016;11(11):1989–98. Epub 2016/08/27. 10.2215/CJN.02110216 27561289 PMC5108188

[23] Ali FN, Hassinger A, Price H, Langman CB. Preoperative plasma FGF23 levels predict acute kidney injury in children: results of a pilot study. Pediatric nephrology (Berlin, Germany). 2013;28(6):959–62. Epub 2013/01/15. 10.1007/s00467-012-2395-2 . 23314442

[24] Speer T, Groesdonk HV, Zapf B, Buescher V, Beyse M, Duerr L, et al A single preoperative FGF23 measurement is a strong predictor of outcome in patients undergoing elective cardiac surgery: a prospective observational study. Critical care (London, England). 2015;19:190 Epub 2015/04/24. 10.1186/s13054-015-0925-6 25902817 PMC4424828

[25] Hanudel MR, Wesseling-Perry K, Gales B, Ramos G, Campbell V, Ethridge K, et al Effects of acute kidney injury and chronic hypoxemia on fibroblast growth factor 23 levels in pediatric cardiac surgery patients. Pediatric nephrology (Berlin, Germany). 2016;31(4):661–9. Epub 2015/11/04. 10.1007/s00467-015-3257-5 26525200 PMC4766020

[26] Leaf DE, Christov M, Juppner H, Siew E, Ikizler TA, Bian A, et al Fibroblast growth factor 23 levels are elevated and associated with severe acute kidney injury and death following cardiac surgery. Kidney international. 2016;89(4):939–48. Epub 2016/03/01. 10.1016/j.kint.2015.12.035 26924052 PMC4801748

[27] Leaf DE, Jacob KA, Srivastava A, Chen ME, Christov M, Juppner H, et al Fibroblast Growth Factor 23 Levels Associate with AKI and Death in Critical Illness. Journal of the American Society of Nephrology: JASN. 2017;28(6):1877–85. Epub 2016/12/29. 10.1681/ASN.2016080836 28028134 PMC5461795

[28] Volovelsky O, Terrell TC, Swain H, Bennett MR, Cooper DS, Goldstein SL. Pre-operative level of FGF23 predicts severe acute kidney injury after heart surgery in children. Pediatric nephrology (Berlin, Germany). 2018;33(12):2363–70. Epub 2018/07/20. 10.1007/s00467-018-4024-1 . 30022312

[29] Nowak KL, Bartz TM, Dalrymple L, de Boer IH, Kestenbaum B, Shlipak MG, et al Fibroblast Growth Factor 23 and the Risk of Infection-Related Hospitalization in Older Adults. Journal of the American Society of Nephrology: JASN. 2017;28(4):1239–46. Epub 2017/01/27. 10.1681/ASN.2016040401 28122946 PMC5373449

[30] Orwoll BE, Sapru A. Biomarkers in Pediatric ARDS: Future Directions. Frontiers in pediatrics. 2016;4:55 Epub 2016/06/18. 10.3389/fped.2016.00055 27313995 PMC4887507

[31] Ventilation with lower tidal volumes as compared with traditional tidal volumes for acute lung injury and the acute respiratory distress syndrome. The Acute Respiratory Distress Syndrome Network. The New England journal of medicine. 2000;342(18):1301–8. 10.1056/NEJM200005043421801 . 10793162

[32] Komaba H, Fukagawa M. The role of FGF23 in CKD—with or without Klotho. Nature reviews Nephrology. 2012;8(8):484–90. Epub 2012/06/21. 10.1038/nrneph.2012.116 . 22714041

[33] Schwartz GJ, Munoz A, Schneider MF, Mak RH, Kaskel F, Warady BA, et al New equations to estimate GFR in children with CKD. Journal of the American Society of Nephrology: JASN. 2009;20(3):629–37. Epub 2009/01/23. 10.1681/ASN.2008030287 19158356 PMC2653687

[34] Akcan-Arikan A, Zappitelli M, Loftis LL, Washburn KK, Jefferson LS, Goldstein SL. Modified RIFLE criteria in critically ill children with acute kidney injury. Kidney international. 2007;71(10):1028–35. 10.1038/sj.ki.5002231 . 17396113

[35] Zinter MS, Spicer AC, Liu KD, Orwoll BE, Alkhouli MF, Brakeman PR, et al Positive Cumulative Fluid Balance Is Associated With Mortality in Pediatric Acute Respiratory Distress Syndrome in the Setting of Acute Kidney Injury. Pediatric critical care medicine: a journal of the Society of Critical Care Medicine and the World Federation of Pediatric Intensive and Critical Care Societies. 2019 Epub 2019/01/24. 10.1097/pcc.0000000000001845 . 30672838 PMC6454886

[36] Pollack MM, Patel KM, Ruttimann UE. PRISM III: an updated Pediatric Risk of Mortality score. Critical care medicine. 1996;24(5):743–52. Epub 1996/05/01. 10.1097/00003246-199605000-00004 . 8706448

[37] Khemani RG, Patel NR, Bart RD 3rd, Newth CJ. Comparison of the pulse oximetric saturation/fraction of inspired oxygen ratio and the PaO2/fraction of inspired oxygen ratio in children. Chest. 2009;135(3):662–8. 10.1378/chest.08-2239 . 19029434

[38] Preacher KJ, Hayes AF. SPSS and SAS procedures for estimating indirect effects in simple mediation models. Behavior research methods, instruments, & computers: a journal of the Psychonomic Society, Inc. 2004;36(4):717–31. Epub 2005/01/12. . 15641418 10.3758/bf03206553

[39] Hayes AF. Beyond Baron and Kenny: Statistical Mediation Analysis in the New Millennium. Communication Monographs. 2009;76(4):408–20. 10.1080/03637750903310360

[40] Fischer DC, Mischek A, Wolf S, Rahn A, Salweski B, Kundt G, et al Paediatric reference values for the C-terminal fragment of fibroblast-growth factor-23, sclerostin, bone-specific alkaline phosphatase and isoform 5b of tartrate-resistant acid phosphatase. Annals of clinical biochemistry. 2012;49(Pt 6):546–53. Epub 2012/09/18. 10.1258/acb.2012.011274 . 22984195

[41] Gkentzi D, Efthymiadou A, Kritikou D, Chrysis D. Fibroblast growth factor 23 and Klotho serum levels in healthy children. Bone. 2014;66:8–14. Epub 2014/06/01. 10.1016/j.bone.2014.05.012 . 24880094

[42] Yamamura M, Yamada Y, Momita S, Kamihira S, Tomonaga M. Circulating interleukin-6 levels are elevated in adult T-cell leukaemia/lymphoma patients and correlate with adverse clinical features and survival. British journal of haematology. 1998;100(1):129–34. Epub 1998/02/05. 10.1046/j.1365-2141.1998.00538.x . 9450801

[43] Farrow EG, Yu X, Summers LJ, Davis SI, Fleet JC, Allen MR, et al Iron deficiency drives an autosomal dominant hypophosphatemic rickets (ADHR) phenotype in fibroblast growth factor-23 (Fgf23) knock-in mice. Proceedings of the National Academy of Sciences of the United States of America. 2011;108(46):E1146–55. Epub 2011/10/19. 10.1073/pnas.1110905108 22006328 PMC3219119

[44] Clinkenbeard EL, Farrow EG, Summers LJ, Cass TA, Roberts JL, Bayt CA, et al Neonatal iron deficiency causes abnormal phosphate metabolism by elevating FGF23 in normal and ADHR mice. Journal of bone and mineral research: the official journal of the American Society for Bone and Mineral Research. 2014;29(2):361–9. Epub 2013/07/23. 10.1002/jbmr.2049 . 23873717 PMC5240191

[45] Hanudel MR, Chua K, Rappaport M, Gabayan V, Valore E, Goltzman D, et al Effects of dietary iron intake and chronic kidney disease on fibroblast growth factor 23 metabolism in wild-type and hepcidin knockout mice. American journal of physiology Renal physiology. 2016;311(6):F1369–f77. Epub 2016/10/14. 10.1152/ajprenal.00281.2016 . 27733366 PMC5210202

[46] Clinkenbeard EL, Hanudel MR, Stayrook KR, Appaiah HN, Farrow EG, Cass TA, et al Erythropoietin stimulates murine and human fibroblast growth factor-23, revealing novel roles for bone and bone marrow. Haematologica. 2017;102(11):e427–e30. Epub 2017/08/19. 10.3324/haematol.2017.167882 28818868 PMC5664401

[47] Rabadi S, Udo I, Leaf DE, Waikar SS, Christov M. Acute blood loss stimulates fibroblast growth factor 23 production. American journal of physiology Renal physiology. 2018;314(1):F132–F9. Epub 2017/09/08. 10.1152/ajprenal.00081.2017 28877877 PMC5866351

[48] Flamme I, Ellinghaus P, Urrego D, Kruger T. FGF23 expression in rodents is directly induced via erythropoietin after inhibition of hypoxia inducible factor proline hydroxylase. PloS one. 2017;12(10):e0186979 Epub 2017/10/27. 10.1371/journal.pone.0186979 29073196 PMC5658123

[49] Toro L, Barrientos V, Leon P, Rojas M, Gonzalez M, Gonzalez-Ibanez A, et al Erythropoietin induces bone marrow and plasma fibroblast growth factor 23 during acute kidney injury. Kidney international. 2018;93(5):1131–41. Epub 2018/02/06. 10.1016/j.kint.2017.11.018 . 29395333

[50] Daryadel A, Bettoni C, Haider T, Imenez Silva PH, Schnitzbauer U, Pastor-Arroyo EM, et al Erythropoietin stimulates fibroblast growth factor 23 (FGF23) in mice and men. Pflugers Archiv: European journal of physiology. 2018;470(10):1569–82. Epub 2018/07/03. 10.1007/s00424-018-2171-7 . 29961920

[51] Hanudel MR, Eisenga MF, Rappaport M, Chua K, Qiao B, Jung G, et al Effects of erythropoietin on fibroblast growth factor 23 in mice and humans. Nephrology, dialysis, transplantation: official publication of the European Dialysis and Transplant Association—European Renal Association. 2018 Epub 2018/07/15. 10.1093/ndt/gfy189 . 30007314 PMC6888066

[52] Knab VM, Corbin B, Andrukhova O, Hum JM, Ni P, Rabadi S, et al Acute Parathyroid Hormone Injection Increases C-Terminal but Not Intact Fibroblast Growth Factor 23 Levels. Endocrinology. 2017;158(5):1130–9. Epub 2017/03/23. 10.1210/en.2016-1451 28324013 PMC5460828

[53] Chawla LS, Seneff MG, Nelson DR, Williams M, Levy H, Kimmel PL, et al Elevated plasma concentrations of IL-6 and elevated APACHE II score predict acute kidney injury in patients with severe sepsis. Clinical journal of the American Society of Nephrology: CJASN. 2007;2(1):22–30. Epub 2007/08/21. 10.2215/CJN.02510706 . 17699383

[54] Greenberg JH, Whitlock R, Zhang WR, Thiessen-Philbrook HR, Zappitelli M, Devarajan P, et al Interleukin-6 and interleukin-10 as acute kidney injury biomarkers in pediatric cardiac surgery. Pediatric nephrology (Berlin, Germany). 2015;30(9):1519–27. Epub 2015/04/17. 10.1007/s00467-015-3088-4 25877915 PMC4537680

[55] Zhang WR, Garg AX, Coca SG, Devereaux PJ, Eikelboom J, Kavsak P, et al Plasma IL-6 and IL-10 Concentrations Predict AKI and Long-Term Mortality in Adults after Cardiac Surgery. Journal of the American Society of Nephrology: JASN. 2015;26(12):3123–32. Epub 2015/04/10. 10.1681/ASN.2014080764 25855775 PMC4657830

[56] Nechemia-Arbely Y, Barkan D, Pizov G, Shriki A, Rose-John S, Galun E, et al IL-6/IL-6R axis plays a critical role in acute kidney injury. Journal of the American Society of Nephrology: JASN. 2008;19(6):1106–15. Epub 2008/03/14. 10.1681/ASN.2007070744 18337485 PMC2396933

[57] Courbebaisse M, Mehel H, Petit-Hoang C, Ribeil JA, Sabbah L, Tuloup-Minguez V, et al Carboxy-terminal fragment of fibroblast growth factor 23 induces heart hypertrophy in sickle cell disease. Haematologica. 2017;102(2):e33–e5. Epub 2016/10/30. 10.3324/haematol.2016.150987 27789679 PMC5286949

[58] Leaf DE, Siew ED, Eisenga MF, Singh K, Mc Causland FR, Srivastava A, et al Fibroblast Growth Factor 23 Associates with Death in Critically Ill Patients. Clinical journal of the American Society of Nephrology: CJASN. 2018;13(4):531–41. Epub 2018/03/10. 10.2215/CJN.10810917 29519954 PMC5969465

[59] Krick S, Baumlin N, Aller SP, Aguiar C, Grabner A, Sailland J, et al Klotho Inhibits Interleukin-8 Secretion from Cystic Fibrosis Airway Epithelia. Scientific reports. 2017;7(1):14388 Epub 2017/11/01. 10.1038/s41598-017-14811-0 29085059 PMC5662572

[60] Krick S, Grabner A, Baumlin N, Yanucil C, Helton S, Grosche A, et al Fibroblast growth factor 23 and Klotho contribute to airway inflammation. The European respiratory journal. 2018;52(1). Epub 2018/05/12. 10.1183/13993003.00236-2018 29748308 PMC6044452

