# Supplementary material for: Plasma total fibroblast growth factor 23 levels are associated with acute kidney injury and mortality in children with acute respiratory distress syndrome
Source: PLoS One. 2019 Sep 5;14(9):e0222065. doi: 10.1371/journal.pone.0222065 (PMC6728039; doi:10.1371/journal.pone.0222065)
Supplement: S1 Table — (DOCX) [file pone.0222065.s001.docx]

**S1 Table.** Numbers of subjects with and without acute kidney injury (AKI).

| **Category** | **Number of Subjects** |
| --- | --- |
| AKI on both Day 1 and Day 3 (persistent AKI) | 24 (15%) |
| No AKI on Day 1, but AKI on Day 3 (incident AKI) | 11 (7%) |
| **Total AKI on Day 3** | **35 (22%)** |
| AKI on Day 1, but no AKI on Day 3 (resolved AKI) | 9 (6%) |
| No AKI on Day 1 or Day 3 (no AKI) | 117 (73%) |
| **Total No AKI on Day 3** | **126 (78%)** |
