# Supplementary material for: Plasma total fibroblast growth factor 23 levels are associated with acute kidney injury and mortality in children with acute respiratory distress syndrome
Source: PLoS One. 2019 Sep 5;14(9):e0222065. doi: 10.1371/journal.pone.0222065 (PMC6728039; doi:10.1371/journal.pone.0222065)
Supplement: S2 Table — All coefficients are adjusted for age, sex, the presence or absence of Day 1 AKI, P/F ratio, and PRISM score. The 95% confidence intervals for the indirect effects were determined after running 10,000 bootstrap samples. The estimated size of the mediated effect (proportion mediated) was calculated as the indirect effect divided by the total effect, multiplied by 100. (DOCX) [file pone.0222065.s002.docx]

**S2 Table.** Mediation analysis of interleukin-6 in the association between total FGF23 and acute kidney injury.

| **Potential mediator** | **Outcome** | **Effect (path)** | **Multivariable model** | | |
| --- | --- | --- | --- | --- | --- |
|  |  |  | **Coefficient (95% CI)** | **p value** | **Proportion mediated** |
| **Interleukin-6** | **Acute kidney injury** | Indirect effect (*ab* path) | 0.012 (-0.004, 0.023) | 0.12 | 36.4% |
|  |  | Total effect (*ab* + *c*’ path) | 0.033 (-0.002, 0.068) | 0.066 |  |

All coefficients are adjusted for age, sex, the presence or absence of Day 1 AKI, P/F ratio, and PRISM score. The 95% confidence intervals for the indirect effects were determined after running 10,000 bootstrap samples. The estimated size of the mediated effect (proportion mediated) was calculated as the indirect effect divided by the total effect, multiplied by 100.
